# Aortic pulse wave velocity, central pulse pressure, augmentation index and chronic kidney disease progression in individuals with type 2 diabetes: a 3- year prospective study

**DOI:** 10.1186/s12882-020-02024-z

**Published:** 2020-08-20

**Authors:** Jian-Jun Liu, Sylvia Liu, Janus Lee, Resham L. Gurung, M. Yiamunaa, Keven Ang, Yi Ming Shao, Robin W. M. Choo, Subramaniam Tavintharan, Wern Ee Tang, Chee Fang Sum, Su Chi Lim

**Affiliations:** 1 Clinical Research Unit, Khoo Teck Puat hospital, Singapore, 768828 Republic of Singapore; 2 Geriatric Education and Research Institute, Singapore, 768024 Republic of Singapore; 3 Diabetes Centre, Admiralty Medical Center, Singapore, 730676 Republic of Singapore; 4 National Healthcare Group Polyclinic, Singapore, 138543 Republic of Singapore; 5 Saw Swee Hock School of Public Heath, National University of Singapore, Singapore, 117549 Republic of Singapore; 6 Diabetes Center, Khoo Teck Puat Hospital, 90 Yishun Central, Singapore, 768828 Republic of Singapore

**Keywords:** Arterial stiffness, Pulse wave velocity, Central pulse pressure, Chronic kidney disease, Type 2 diabetes

## Abstract

**Background:**

Pulse wave velocity (PWV), central pulse pressure and augmentation index are arterial stiffness- related hemodynamic parameters but their associations with renal outcome are still controversial. We hereby aim to study, 1) which hemodynamic parameter is independently associated with progressive chronic kidney disease (CKD), 2) the association of 3-year change in PWV with CKD progression and, 3) the additive predictive value of PWV for progressive CKD.

**Methods:**

Carotid- femoral PWV, central pulse pressure and augmentation index were measured in 1444 participants with type 2 diabetes at baseline and 3 years apart. Progressive CKD was defined as confirmed eGFR decline 40% or greater.

**Results:**

In the follow-up, 102 participants experienced progressive CKD. All 3 hemodynamic parameters were significantly associated with progressive CKD In univariable analysis. However, only PWV remained statistically significant after adjustment for known clinical risk factors and the other 2 hemodynamic parameters (OR 1.14 [95% CI 1.01–1.29] per m/s increment). One m/s regression (decrement) in PWV in the 3-year follow-up was associated with 26% lower adjusted- risk of progressive CKD (OR 0.74, 95% CI 0.56–0.97). Adding PWV onto traditional risk factor- based model significantly improved classification (net reclassification improvement 0.25, 95% CI 0.05–0.45, *P* = 0.01) and positive prediction rate (24.5 to 32.3%).

**Conclusions:**

Of 3 arterial stiffness- related hemodynamic parameters, only PWV is independently associated with progressive CKD. PWV may be a potential intervention target to mitigate risk of CKD progression and also a biomarker to improve risk-stratification of adverse renal outcome in individuals with type 2 diabetes.

## Background

Diabetic kidney disease affects more than 30% of patients with type 2 diabetes. It is not only the leading cause of end stage renal disease in developed countries but also an independent risk factor for cardiovascular disease and mortality [1, 2]. Both metabolic risk factors and hemodynamic dysregulations are involved in the development and progression of chronic kidney disease (CKD) in patients with diabetes [3]. Accumulating data suggest that arterial stiffness, an established cardiovascular risk factor, plays a causal role in pathogenesis of CKD [4]. The underpinning mechanisms are thought to involve an increased penetration of energy within the pulse wave into the high flow but low resistance kidney, leading to injury on glomerular capillaries [5, 6].

Aorta is the recommended site and pulse wave velocity (PWV) is the most widely used technique to assess arterial stiffness and its relationship with disease outcomes [7, 8]. A vast number of early studies have associated an increased PWV with declining kidney function in both diabetic and non-diabetic populations [9–11]. Neverthelss, several studies did not observe an independent association between PWV and adverse renal outcomes [12–16], suggesting that more studies are warranted. To our knowledge, literature on the relationship between PWV and renal outcome in Asian population are still scarce. On the other hand, an increased PWV also amplifies aortic pressures during systole, reduces pressure during diastole and consequently elevates central pulse pressure [17, 18]. Intriguingly, an early study found that central pulse pressure, but not PWV, was associated with progression to end stage renal disease in patients with CKD [12]. Whether this finding can be observed in diabetic population with a broad spectrum of kidney function remains unknown. In addition to PWV and central pulse pressure, the impedance mismatch due to aortic geometry changes and local arterial branching causes partial reflection of forward pressure waves traveling back to the central aorta. The magnitude of wave reflection quantified by augmentation index is determined by several factors related to, but not limited to arterial stiffness [7, 19]. To our knowledge, the interrelationship of aortic PWV, central pulse pressure and augmentation index have not been characterized and the strength of their associations with renal outcomes as compared to one another has not been studied.

The other knowledge gap regarding the role of arterial stiffness in kidney disease is that most early studies focused on baseline PWV level and subsequent kidney disease outcome in the follow-up. Data on dynamic change in arterial stiffness, which requires repeated measurements, and renal outcome is still lacking, especially in diabetic population. Additionally, the value of PWV as a biomarker for prediction of renal outcome above and beyond traditional risk factors has not been systematically assessed although its association with declining kidney function has been demonstrated in several early studies [11, 20].

In current work, we aim to study, 1) which of the 3 hemodynamic parameters, i.e. PWV, central pulse pressure and augmentation index, has the strongest association with progressive CKD; 2) whether the 3- year change in PWV is associated with risk of CKD progression; 3) the additive value of PWV as a biomarker for prediction of progressive CKD above traditional clinical risk factors in individuals with type 2 diabetes.

## Participants and methods

Details of SMART2D (Singapore Study of Macro-angiopathy and Micro-Vascular Reactivity in Type 2 Diabetes) cohort have been described elsewhere [21, 22]. In brief, 2057 outpatients with type 2 diabetes were recruited from a secondary hospital and a nearly primary care medical facility in the northern region of Singapore between August 2011 and March 2014. Exclusion criteria included pregnancy, point-of-care fasting glucose > 15.0 mM or < 4.5 mM, HbA1c > 12% (108 mmol/mol), autoimmune disease and cancer on active treatments. Participants taking steroid equivalent to 5 mg prednisolone on the day of data and sample collection were also excluded.

Three years after enrolment date, all participants were invited consecutively by written mail and phone call for the planned 3-year follow-up visit in the hospital. By Aug 2018, 1510 participants have completed the follow-up visit. We excluded 23 participants with baseline eGFR ≤15 ml/min/1.73m^2^ (including 11 on dialysis), 23 with no arterial stiffness measurement at baseline and 20 participants with no arterial stiffness assessment at follow-up visit. A total of 1444 participants were included in the final analysis.

### Definition of progressive chronic kidney disease

Progressive CKD was defined as eGFR decline 40% or greater from baseline to the time of 3- year follow-up visit. This endpoint was recommended as a valid surrogate for hard renal outcomes, i.e. doubling of serum creatinine and end stage renal disease, by Food and Drug Agency and US National Kidney Foundation [23, 24]. To exclude rapid renal function decline attributable to acute kidney disease, the 40% eGFR decline was confirmed by a separate eGFR reading in the electronic medical record 3 months apart the follow-up visit date.

Additionally, we defined progressive CKD as doubling of serum creatinine from baseline to the 3-year follow-up visit as a sensitivity analysis.

### Carotid- femoral pulse wave velocity measurement and waveform analysis

Hemodynamic parameter measurement was performed in a quiet room with controlled temperature. Participant rested in supine position for 10 min before the assay procedure. Carotid-femoral PWV was measured by the foot- to- foot method (SphygmoCor, AtCor Medical, Sydney, Australia). Briefly, the transit time of the waves between carotid and femoral sites was measured by sequential tonometry gated by electrocardiogram. PWV was expressed as carotid- femoral distance (meter) divided by transit time (second) [25]. Given that the direct carotid femoral distance is about 20% longer than the actual distance travelled by pulse wave, we adjusted PWV by a scaling factor of 0.8 as recommended by European Society of Hypertension and European Society of Cardiology [8, 26]. Both baseline and follow-up PWV were measured by the same 3 trained operators. The operators were blinded to results of baseline measurement during the follow-up examination. Given that measurement of wave travel distance was the major source of inaccuracy, the carotid-femoral distance measured at baseline was used to calculate PWV in the second examination at the follow-up visit [27]. The change in PWV (m/s) was calculated as absolute change, i.e. the second measurement minus the first one [27]. The same group of 3 research nurses performed the haemodynamic measurements for both baseline and follow-up studies. Based on assessment of performances on 4 randomly selected participants, the intra- and inter- operator coefficient of variations for repeated PWV measurements were 5.8 and 7.2%, respectively [28].

Central blood pressure and augmentation index were derived from pressure waveform analysis. Measurements were performed by placing the tonometer over the radial artery and recording 11 s of waveforms by the SphygmoCor system.

### Clinical and biochemical variables

Peripheral blood pressure and resting heart rate were measured 3 times in a sitting position with 5- min interval by a semi-automatic sphygmomanometer and the average of 3 readings was used. Mean arterial pressure (MAP) was calculated as (systolic blood pressure + 2 X diastolic blood pressure)/3. Hypertension was defined as receiving any of 4 classes of anti-hypertension medications (calcium blocker, renin angiotensin system blocker, diuretics and beta-blocker) or blood pressure above 140/90 mmHg at cohort enrolment. Atrial fibrillation was identified by 20 beats electrocardiogram at PWV measurement by SphygmoCor. Medication usage was retrieved from electronic medical record and medication dispensary database. Ethnicity, smoking status and duration of diabetes were self-reported. Fasting plasma glucose, serum triacylglycerol, HDL and LDL cholesterol were measured by enzymatic methods (Roche Cobas Integra 700, Roche Diagnostics, Swiss). HbA1c was quantified by a point-of-care immunoassay analyzer (DCA Vantage Analyzer, Siemens, Germany). Urinary albumin was measured by a solid phase competitive chemiluminescent immunoassay (Immulite, DPC, Gwynedd, UK). Creatinine was measured by an enzymatic method which was traceable to isotope dilution mass spectrometry reference. The eGFR was calculated by the CKD- Epidemiology Collaboration formula. Both baseline and follow-up biochemical assays were performed in the same central lab.

### Data analysis

Data were presented as mean ± standard deviation (SD), median (interquartile range, IQR) or proportion as appropriate. Differences in clinical and biochemical variables across PWV tertiles were compared by one way ANOVA, Kruskal- Wallis test or χ2 test. Comparison of two- group differences was performed by student t test, Mann- Whitney U test or χ2 test.

We employed multivariable logistic regression to study the association of PWV, central pulse pressure and augmentation index with progressive CKD. Progressive CKD (yes or no) was dependent variable. Covariates were selected a priori based on biological plausibility. Age was one of the main determinants of arterial stiffness [29]. The most significant physiological variable affecting arterial stiffness is vessel distending pressure, i.e. MAP [7]. Hence, both age and MAP were included as covariates as recommended [7]. There is a strong relationship between heart rate and PWV [28, 30]. Therefore, we included resting heart rate as one of the main confounders. For this reason, we did not correct augmentation index to 75 heart beat per minute in multivariable analysis. Additionally, we included the following covariates in the model: sex, ethnicity (Chinese as reference), smoking status (active smoker versus others), duration of diabetes, body mass index, HbA1c, HDL, LDL cholesterol, triacylglycerol (log-transformed), usage of insulin, renin-angiotensin system blocker, baseline eGFR and urinary albumin-to-creatinine ratio (ACR, log-transformed), two of the most important determinants of progressive CKD. Usage of statin, calcium blocker, diuretics and beta blocker might be confounding by indication. We did not include these medications in the model because we adjusted lipids profile and blood pressure. Of 1444 participants in the current study, only 7 were identified to have atrial fibrillation at baseline and 4 had incident atrial fibrillation in follow-up visit. Hence, we did not include this risk factor in data analysis due to the small numbers.

We used C-statistics and continuous net reclassification improvement (cNRI) to assess the additive value of PWV for prediction of progressive CKD above and beyond traditional risk factors. Because of the low frequency of renal outcome, the negative prediction rate was high but positive predictive rate was low in clinical trials and cohort studies [24, 31]. Hence, we studied whether adding PWV onto traditional risk factors may improve positive predictive rate. To improve potential clinical application, we first used forward logistic regression to select a parsimonious panel of clinical variables which independently predicted progressive CKD, followed by identification of cut-points using the classification and regression tree approach in rpart package in R software. This method involves hierarchical partitioning of the study group on the basis of optimal cut-points in the distribution of each prediction variable [32].

Statistical analysis was performed by SPSS (version 22) and R software (version 3.4.2). A 2- sided *P* < 0.05 was considered as significant for all analyses.

## Results

### Participant baseline characteristics

Participants with loss to follow-up were older, had a higher blood pressure and aortic stiffness, and more likely on anti-hypertension medications. In addition, they had poorer kidney function at baseline and more likely to be Malay ethnicity (Additional file 1, supplementary Table 1).

The average age of participants in the current analysis was 57.7 (SD 10.3) years old, diabetes duration was 10.9 (SD 8.6) years and 51.7% were male. Baseline characteristics were visualized after stratifying participants according to tertiles of PWV (Table 1). Not unexpected, participants with PWV in high tertile were older, had a longer duration of diabetes, higher HbA1c and blood pressure. They also had a lower eGFR, higher level of albuminuria, and more likely on statin, insulin and anti-hypertension medication treatments.

**Table 1:** Baseline clinical and biochemical characteristics stratified by PWV tertiles

	All participants *N* = 1444	Tertile 1 *N* = 482	Tertile 2 *N* = 481	Tertile 3 *N* = 481	*P* value *
Pulse wave velocity (m/s)	7.7 ± 2.1	5.6 ± 0.7	7.3 ± 0.5	10.0 ± 1.7	–
Index age (years)	57.7 ± 10.3	52.1 ± 10.7	57.0 ± 9.6	60.8 ± 8.6	< 0.001
Male sex (%)	51.7	51.7	50.3	53.2	0.66
Ethnicity (%)					0.30
Chinese	53.7	53.5	52.0	55.5	
Malay	20.1	17.8	22.0	20.6	
Asian Indian	26.2	28.6	26.0	23.9	
Diabetes duration (years)	10.9 ± 8.6	7.9 ± 6.9	10.9 ± 8.6	13.8 ± 9.1	< 0.001
CVD history (%)	6.8	5.2	8.1	7.1	0.19
Current smoker (%)	8.5	10.8	7.3	7.5	0.09
Body mass index (kg/m2)	27.7 ± 5.1	27.4 ± 5.0	27.8 ± 5.1	27.8 ± 5.1	0.28
Fasting glucose (mM)	8.1 ± 2.6	7.8 ± 2.5	8.1 ± 2.5	8.2 ± 2.6	0.06
HbA1c (%)	7.8 ± 1.3	7.5 ± 1.3	7.9 ± 1.3	7.9 ± 1.3	< 0.001
Resting Heart rate (bpm)	71 ± 11	70 ± 10	71 ± 11	71 ± 11	0.04
Blood pressure (mmHg)					
Systolic pressure	139 ± 18	130 ± 15	139 ± 17	146 ± 18	< 0.001
Diastolic pressure	79 ± 9	78 ± 9	79 ± 10	79 ± 9	0.05
Mean Arterial Pressure	99 ± 10	96 ± 9	99 ± 11	101 ± 10	< 0.001
Lipids profile (mM)					
HDL Cholesterol	1.30 ± 0.36	1.30 ± 0.35	1.29 ± 0.37	1.29 ± 0.36	0.93
LDL Cholesterol	2.75 ± 0.81	2.83 ± 0.83	2.73 ± 0.83	2.67 ± 0.78	0.01
Triacylglycerol (IQR)	1.38 (1.02–1.92)	1.34 (0.96–1.90)	1.40 (1.06–1.93)	1.42 (1.05–1.92)	0.09
Central PP (mmHg)	49 ± 16	42 ± 14	48 ± 15	55 ± 16	< 0.001
Central PP > 50 mmHg (%)	41.6	25.3	41.7	58.5	< 0.001
Augmentation index (%)	26 ± 11	24 ± 11	26 ± 11	28 ± 10	< 0.001
Baseline renal function					
eGFR (ml/min/1.73m^2^)	89 ± 24	97 ± 19	89 ± 23	80 ± 25	< 0.001
ACR (μg/mg)	21 (6.0–85)	11 (4.0–36)	22 (7.0–81)	38 (11.0–237)	< 0.001
Medications usage (%)					
Statin	81.0	78.4	79.1	85.4	0.01
Insulin	26.6	18.2	25.1	36.5	< 0.001
RAS blocker	59.4	43.4	63.3	71.2	< 0.001
Calcium channel blocker	19.8	10.6	20.6	28.5	< 0.001
Beta blocker	14.2	7.5	16.2	19.1	< 0.001
Diuretics	12.8	4.8	12.3	21.4	< 0.001
Hypertension on- treatment	84.4	81.1	83.7	87.3	0.07

* one way ANOVA, Kruskal- Wallis test or χ2 test where appropriate. *PP* pulse pressure, *RAS* renin- angiotensin system, *ACR* albumin-to-creatinine ratio

Participants with a higher PWV also had higher central pulse pressure and augmentation index. PWV, augmentation index and central pulse pressure were only modestly or moderately correlated (Pearson *r* = 0.15 for PWV and augmentation index; *r* = 0.37 for PWV and central pulse pressure; *r* = 0.41 for central pulse pressure and augmentation index) with one another at baseline.

### Associations of PWV, central pulse pressure and augmentation index with progressive CKD

At the 3- year follow-up, 102 participants experienced progressive CKD and all were confirmed by a separate eGFR measurement 3 months apart from the follow-up visit. Participants with progressive CKD had a longer diabetes duration, higher HbA1c, blood pressure, triacylglycerol, albuminuria and a lower eGFR. They were less likely to be Asian Indian ethnicity, more likely to be active smoker and more likely on anti-hypertensive medication treatment. Noteworthy, those with progressive CKD had significantly higher PWV, central pulse pressure and augmentation at baseline (Additional file 1, supplementary Table 2).

In univariable analysis, all 3 hemodynamic parameters were significantly associated with progressive CKD. Adjustment for multiple demographic confounders and cardio-renal risk factors including baseline eGFR and urinary ACR attenuated the strength of association between PWV and progressive CKD (OR 1.32 [95% CI 1.22–1.43] to 1.13 [95% CI 1.01–1.27] per one m/s increment) and also weakened the association between central pulse pressure and progressive CKD (OR 1.39 [95% CI 1.23–1.56] to 1.23 [95% CI 0.99–1.52] per 10 mmHg increment). The point estimate of association between augmentation index and progressive CKD did not change materially in multivariable model but the confidence interval became broader (OR 1.33 [95% 1.11–1.60] to 1.31 [95% CI 1.00–1.72], Table 2).

**Table 2:** Association of PWV, central pulse pressure and augmentation index with progressive CKD

	Progression Defined as 40% eGFR Decline			Progression Defined as Doubling of Serum Creatinine		
	Univariable (Model 1)	Multivariable (Model 2)	Multivariable + other 2 hemodynamic parameters (Model 3)	Univariable (Model 1)	Multivariable (Model 2)	Multivariable + other 2 hemodynamic parameters (Model 3)
**PWV (m/s)**	1.32 (1.22–1.430) *P* < 0.001	1.13 (1.01–1.27) *P* = 0.04	1.14 (1.01–1.29) *P* = 0.04	1.35 (1.23–1.49) *P* < 0.001	1.18 (1.02–1.36) *P* = 0.02	1.19 (1.02–1.39) *P* = 0.03
**Central Pulse Pressure (10 mmHg)**	1.39 (1.23–1.56) *P* < 0.001	1.23 (0.99–1.52) *P* = 0.06	1.14 (0.91–1.43) *P* = 0.27	1.28 (1.09–1.49) *P* = 0.002	1.13 (0.86–1.49) *P* = 0.38	1.05 (0.78–1.41) *P* = 0.74
**Augmentation Index (10%)**	1.33 (1.11–1.60) *P* = 0.001	1.31 (1.00–1.72) *P* = 0.049	1.22 (0.92–1.63) *P* = 0.16	1.31 (1.03–1.66) *P* = 0.03	1.25 (0.90–1.73) *P* = 0.19	1.19 (0.83–1.67) *P* = 0.35

Binary logistic regression model- Progressive CKD (yes or no) as outcome. Multivariable model adjusted age, sex, ethnicity (Chinese as reference), smoking (active smoker versus others), duration of diabetes, body mass index, HbA1c, mean arterial pressure, resting heart rate, HDL and LDL cholesterol, triacylglycerol (log-transformed), baseline eGFR, urinary albumin-to-creatinine ratio (log-transformed), usage of insulin (yes or no), and renin-angiotensin system blocker (yes or no)

In the multivariable model with all 3 hemodynamic parameters included, only PWV remained significantly associated with progressive CKD (OR 1.14 [95% CI 1.01–1.29] per one m/s increment). Neither central pulse pressure nor augmentation index were significantly associated with CKD progression after adjustment for known clinical risk factors and the other 2 hemodynamic parameters (Table 2).

In sensitivity analysis, we identified 55 participants with serum creatinine level doubling at the 3- year follow-up. Similar to the primary analysis, only PWV was significantly associated with risk of doubling of serum creatinine in multivariable analysis (Table 2).

### Regression of PWV in the 3-year follow-up was associated with a lower risk of progressive CKD

Participants with PWV regression ([follow-up PWV – baseline PWV] ≤ 0 m/s) were older, had a higher baseline PWV, were more likely on renin-angiotensin system blocker treatment but less likely to be active smoker. There were no significant differences in other demographic and cardio-renal risk factors between those with PWV regression and progression (Additional file 1, supplementary Table 3).

In 729 participants with PWV regression, 55 (7.5%) experienced CKD progression whilst in 715 participants with PWV progression, 47 (6.6%) experienced CKD progression (*P* = 0.47). As compared to those with PWV progression, participants with PWV regression was associated with a statistically non-significant but numerically lower risk of progressive CKD after accounting for baseline PWV level (OR 0.69 [95% CI 0.44–1.07], *P* = 0.10).

Further exploratory analysis after stratifying participants according to PWV regression versus progression suggested that 1 m/s PWV decrement in the 3-year follow-up was associated with 26% lower risk of progressive CKD (OR 0.74, 95% CI 0.56–0.97, *P* = 0.03) after adjustment for baseline PWV and known clinical risk factors (all covariates in model 2, Table 2). On the other hand, in participants with PWV progression, the change in PWV in the 3-year follow-up duration was not significantly associated with risk of progressive CKD (adjusted OR 1.01, 95% CI 0.77–1.33 per 1 m/s increment, *P* = 0.94).

### Additive value of PWV as a biomarker for prediction of progressive CKD above traditional risk factors

Forward logistic regression suggested that ethnicity, HbA1c, MAP, eGFR and ACR were independent predictors of progressive CKD. Given that age was one of the main determinants of PWV, we also included this variable in the model. Adding PWV onto the traditional risk factor- based model significantly increased cNRI (0.25, 95% CI 0.05–0.45, *P* = 0.01) but did not improve C statistics (AUC 0.887 versus 0.892, *P* = 0.84, Table 3).

**Table 3:** Additive value for prediction of progressive CKD by PWV

	Clinical model ^**a**^	Clinical model + PWV	***P*** value
**AUC**	0.887	0.892	0.84
**cNRI (95% CI)**	reference	0.25 (0.05–0.45)	0.01
**Positive prediction rate (%)** ^**b**^	24.5	32.3	–
**Negative prediction rate (%)** ^**b**^	99.2	98.3	–

^a^ Clinical model included age, ethnicity (Chinese as reference), HbA1c, MAP, eGFR and ACR; ^b^ PWV was dichotomized as > 8.6 m/s versus ≤8.6 m/s when being added into clinical model for estimation of positive and negative prediction rates

Rpart package suggested urinary ACR > 470 mg/g, eGFR < 33 ml/min/1.73m^2^, MAP > 110 mmHg, HbA1c > 8.5% and PWV > 8.6 m/s as the optimal cut- points to produce the widest separation of CKD progressors versus non-progressors in the study cohort. Adding PWV onto the traditional risk factor- based model increased positive prediction rate from 24.5 to 32.3% without markedly compromising negative prediction rate (99.2 to 98.3%, Table 3).

## Discussions

In this prospective cohort study, the main findings include, 1) of the 3 arterial stiffness- related hemodynamic parameters, only PWV, but not augmentation index and central pulse pressure, was independently associated with progressive CKD after accounting for known clinical risk factors and adjustment for the other 2 hemodynamic parameters; 2) regression of PWV in the 3-year follow-up was associated with a reduced risk of CKD progression; 3) adding PWV onto traditional risk factor- based model improved net reclassification and positive prediction rate of progressive CKD. These findings suggest that PWV may be explored as not only a biomarker for better risk-stratification of adverse renal outcome but also a potential intervention target to mitigate the risk of progressive CKD in individuals with type 2 diabetes.

The independent association of PWV with risk of progressive CKD observed in our diabetic population in South East Asia is agreeable with several previous studies. Nevertheless, we extended the early literature by showing that PWV has the strongest association with progressive CKD among the 3 distinct but overlapping hemodynamic parameters. This notion is supported by the finding that the associations of central pulse pressure and augmentation index with risk of progressive CKD were markedly attenuated after adjustment for PWV whereas the association of PWV with progressive CKD did not materially change after adjustment for the other 2 hemodynamic parameters in the multivariable model (Table 2). These data prompted us to explore whether the 3-year change in PWV was associated with risk of adverse renal outcome and whether PWV has additive value for prediction of CKD progression above traditional risk factors. To our knowledge, these two questions have not been systematically addressed before.

The mechanism by which a high PWV leads to CKD progression is likely attributable to an increment in propagation of energy in pulsatile flow wave into the low resistance kidney, especially in patients with diabetes [5, 6]. This is because diabetic kidney is susceptible to loss of the protective autoregulation on blood flow which results in exacerbation of the pulsatile energy transmission, damage of glomerular vasculature and progressive loss of kidney function [17, 33]. PWV has been proposed as potential intervention target to improve prognosis of cardiovascular disease but data regarding whether targeting PWV may also confer beneficial effect on the prognosis of renal outcome are scarce [34]. In line with the discussion above, our current study showed that regression of PWV within the 3-year follow-up was associated with a reduced risk of progressive CKD. This finding suggests the need to quest after strategies to specifically target arterial stiffness in future studies. On the other hand, medication treatments in diabetic patients should also consider the beneficial effect on arterial stiffness. For example, pharmacological inhibition of angiotensin II signalling may translate into a reduced arterial stiffness [35, 36]. Interestingly, our data showed that participants with PWV regression were more likely on renin-angiotensin system inhibitor treatment despite having no significant difference in albuminuria at baseline (Additional file 1, supplementary Table 3). Other medications with renal protective effects including SGLT2 inhibitors and non- medication modalities such as physical exercise also demonstrated beneficial effects on arterial stiffening [37, 38]. It is therefore reasonable to postulate that renal protective effect of SGLT2 inhibitors and physical exercise may be partly attributable to their actions on arterial stiffness. Intriguingly, we did not observe a significant association between PWV progression in the follow-up and an increased risk of progressive CKD in the 3-year interval. The reasons remain to be elucidated but we speculate that an increased PWV may take a longer time to exert its deleterious effect on kidney ultrastructure and filtration function whereas regression in PWV will lead to a rapid intrarenal hemodynamic change with manifestation of a reduced risk of progressive CKD in a short period. Nevertheless, we would like to highlight that these findings are exploratory in nature and future external validations and mechanistic studies are warranted.

Clinically accessible biomarkers predictive of adverse renal outcomes may aid stratification of patients for early intervention to prevent or mitigate risk of progressive kidney disease [39]. To our knowledge, the current work is probably the first to assess the additive value of PWV for prediction of renal outcome above and beyond traditional risk factors. Given that only a small proportion of patients will experience progressive CKD and reach the hard renal outcomes, i.e. doubling of serum creatinine and/or end stage renal disease [24], the challenge in risk prediction of renal outcome lies in the low positive prediction rate among at- risk patients [31]. Our analysis showed that PWV as a biomarker may improve classification despite modest increment in C- statistics. More importantly, adding PWV onto traditional risk factor- based model may enhance positive prediction rate. These findings support that, besides its established application as a valid biomarker for cardiovascular risk [26], PWV may be potentially be a biomarker to aid risk-stratification for individualized management of diabetic patients and enrich participants with high renal risk for enrolment in clinical trials.

The current work has several strengths. Participant baseline phenotype was carefully characterized and the planned 3-year follow-up study was performed by the same group of researchers. Confirmed 40% eGFR decline is a valid surrogate renal outcome recommended by leading organizations which has been assessed in large studies [23, 24]. The methodology for assessment of arterial stiffness by measurement of carotid- femoral PWV is a well- established technique to study the relationship between arterial stiffness and disease outcomes [7, 8, 26]. In addition, we have considered multiple known demographic and cardio-renal risk factors in our analysis. Nevertheless, some important weaknesses should be mentioned. First, as for all observational studies, residual confounding is inevitable even though we have adjusted the known clinical risk factors. We cannot infer causality based on our findings either. The study on the relationship between dynamic change in PWV and risk of progressive CKD can only be taken as an exploratory analysis for hypothesis generation. Second, the phenomenon of “regression to the mean” is a potential confounder for all studies with repeated measurements. However, the correlation coefficient between the first and second measurements of PWV was relatively high (*r* = 0.55) which may partly decrease the regression to the mean [40]. Also, we have adjusted baseline value of PWV in all our analyses which is an acceptable approach to reduce regression to the mean phenomenon [27, 40]. Third, our participants were South East Asians. External validation in other ethnic groups is warranted to assess the generalizability of our findings. In addition, it is unknown whether our findings can be extrapolated to non-diabetic populations either. Finally, the study involved two aortic haemodynamic measurements 3 years apart. Participants with worsening conditions and progressive disease may be more likely lost to follow-up at the second visit (Additional file 1, supplementary Table 1). This inherent weakness of the study may limit generalizability of our findings.

## Conclusion

Both baseline PWV and its 3-year change were independently associated with risk of progressive CKD, lending evidence to support that the effect on arterial stiffness should be considered in management of diabetes and its complications in individuals with type 2 diabetes. Moreover, PWV improved reclassification and positive prediction rate for risk of progressive CKD above and beyond traditional risk factors. Future studies are warranted to examine whether PWV may be taken as a novel biomarker for risk-stratification of diabetic patients in terms of adverse renal outcome or aid the enrichment of high risk patients to improve the efficiency of clinical trials with renal outcomes.

## Supplementary information

**Additional file 1.** Supplementary Table 1, 2 and 3.

## Data Availability

The datasets generated and analysed during the current study are not publicly available due to ethical restrictions. However, anonymized or aggregated data are available from the corresponding author on reasonable request and upon approval from Singapore National Healthcare Group Review Board.

## Abbreviations

ACR: Albumin-to-creatinine ratio
CKD: Chronic kidney disease
cNRI: Continuous net reclassification improvement
eGFR: Estimated glomerular filtration rate
MAP: Mean arterial pressure
PWV: Pulse wave velocity
