# Supplementary material for: Aortic pulse wave velocity, central pulse pressure, augmentation index and chronic kidney disease progression in individuals with type 2 diabetes: a 3- year prospective study
Source: BMC Nephrol. 2020 Aug 20;21:359. doi: 10.1186/s12882-020-02024-z (PMC7441695; doi:10.1186/s12882-020-02024-z)
Supplement: Supplementary file 1 — Additional file 1. Supplementary Table 1, 2 and 3. [file 12882_2020_2024_MOESM1_ESM.docx]

**Supplementary Table 1: Baseline clinical and biochemical characteristics of participants with and without the 3-year follow-up**

|  | With follow-up  N=1510 | Loss to follow-up  N=547 | P value * |
| --- | --- | --- | --- |
| Index age (years) | 56.7 ± 10.4 | 59.1 ± 11.8 | <0.01 |
| Male sex (%) | 51.8 | 48.6 | 0.21 |
| Ethnicity (%)  Chinese  Malay  Asian Indian | 53.5  20.3  26.2 | 44.8  28.7  26.5 | <0.01 |
| Diabetes duration (years) | 11.0 ± 8.7 | 12.2 ± 10.1 | 0.01 |
| Current smoker (%) | 8.6 | 8.8 | 0.87 |
| CVD history (%) | 6.9 | 9.5 | 0.05 |
| Body mass index (kg/m2) | 27.8 ± 5.3 | 27.6 ± 5.2 | 0.61 |
| Fasting glucose (mM) | 8.1 ± 2.6 | 8.2 ± 2.7 | 0.37 |
| HbA1c (%) | 7.8 ± 1.3 | 7.9 ± 1.4 | 0.11 |
| Heart rate (bpm) | 71 ± 11 | 72 ± 11 | 0.04 |
| Blood pressure (mmHg)  Systolic pressure  Diastolic pressure  Mean Arterial Pressure | 139 ± 18  79 ± 9  99 ± 11 | 147 ± 21  80 ± 10  102 ± 12 | <0.01  0.06  <0.01 |
| Lipids profile (mM)  HDL Cholesterol  LDL Cholesterol  Triacylglycerol (IQR) | 1.29 ± 0.36  2.75 ± 0.81  1.39 (1.03-1.94) | 1.29 ± 0.34  2.80 ± 0.90  1.44 (1.10-2.01) | 0.71  0.24  0.10 |
| Pulse wave velocity (m/s) | 7.7 ± 2.2 | 8.1 ± 2.4 | <0.01 |
| Central PP (mmHg)  Central PP > 50 mmHg (%) | 49 ± 17  42.2 | 54 ± 20  53.7 | <0.001  <0.01 |
| Augmentation index (%) | 26 ± 11 | 27 ± 10 | 0.02 |
| Baseline renal function  eGFR (ml/min/1.73m^2^)  ACR (µg/mg) IQR | 87 ± 22  22 (6.0-89) | 78 ± 32  34 (9.0-211) | <0.01  <0.01 |
| Medications usage (%)  Statin  Insulin  RAS blocker  Calcium channel blocker  Beta blocker  Diuretics | 80.9  27.3  59.6  20.9  15.4  13.9 | 81.1  32.7  62.0  27.2  24.5  20.7 | 0.93  0.02  0.33  0.02  <0.01  <0.01 |

* Student t test, Mann-Whitney U test or χ2 test where appropriate. PP, pulse pressure; ACR, urinary albumin-to-creatinine ratio; RAS, renin-angiotensin system

**Supplementary Table 2: Participant baseline clinical and biochemical characteristics stratified by progressive CKD**

|  | Non-progressor  N=1342 | CKD progressors  N=102 | P value * |
| --- | --- | --- | --- |
| Index age (years) | 56.7 ± 10.3 | 56.6 ± 10.4 | 0.92 |
| Male sex (%) | 51.0 | 60.8 | 0.06 |
| Ethnicity (%)  Chinese  Malay  Asian Indian | 53.4  19.2  27.5 | 57.8  32.4  9.80 | <0.001 |
| Diabetes duration (years) | 10.7 ± 8.5 | 13.2 ± 9.3 | 0.01 |
| Current smoker (%) | 8.1 | 13.7 | 0.05 |
| CVD history (%) | 6.8 | 6.9 | 0.98 |
| Body mass index (kg/m2) | 27.6 ± 5.1 | 28.2 ± 4.6 | 0.27 |
| Fasting glucose (mM) | 8.0 ± 2.5 | 9.2 ± 3.0 | <0.001 |
| HbA1c (%) | 7.7 ± 1.3 | 8.4 ± 1.5 | <0.001 |
| Heart rate (bpm) | 70 ± 11 | 74 ± 11 | 0.001 |
| Blood pressure (mmHg)  Systolic pressure  Diastolic pressure  Mean Arterial Pressure | 138 ± 17  79 ± 9  98 ± 10 | 154 ± 19  83 ± 9  107 ± 10 | <0.001  <0.001  <0.001 |
| Lipids profile (mM)  HDL Cholesterol  LDL Cholesterol  Triacylglycerol (IQR) | 1.30 ± 0.36  2.74 ± 0.81  1.37 (1.02-1.90) | 1.26 ± 0.34  2.81 ± 0.84  1.62 (1.20-2.47) | 0.35  0.45  <0.001 |
| Pulse wave velocity (m/s) | 7.6 ± 2.1 | 9.2 ± 2.4 | <0.001 |
| Central PP (mmHg)  Central PP > 50 mmHg (%) | 48 ± 15  39.9 | 57 ± 18  64.5 | <0.001  <0.001 |
| Augmentation index (%) | 26 ± 11 | 29 ± 12 | 0.002 |
| Baseline renal function  eGFR (ml/min/1.73m^2^)  ACR (µg/mg) IQR | 90 ± 22  18 (6.0-64) | 67 ± 31  626 (206-1918) | <0.001  <0.001 |
| Medications usage (%)  Statin  Insulin  RAS blocker  Calcium channel blocker  Beta blocker  Diuretics | 80.3  24.6  57.6  17.5  13.3  10.6 | 89.2  52.5  82.4  50.0  26.5  42.2 | 0.03  <0.001  <0.001  <0.001  <0.001  <0.001 |

* Student t test, Mann-Whitney U test or χ2 test where appropriate. PP, pulse pressure; ACR, urinary albumin-to-creatinine ratio; RAS, renin-angiotensin system

**Supplementary Table 3: Participant baseline clinical and biochemical characteristics stratified by PWV change in 3-year follow-up**

|  | PWV progression  N=715 | PWV regression  N=729 | P value * |
| --- | --- | --- | --- |
| Index age (years) | 56.0 ± 10.3 | 57.3 ± 10.3 | 0.02 |
| Male sex (%) | 51.6 | 51.9 | 0.93 |
| Ethnicity (%)  Chinese  Malay  Asian Indian | 55.1  20.0  24.9 | 52.3  20.3  27.4 | 0.49 |
| Diabetes duration (years) | 10.5 ± 8.4 | 11.3 ± 8.8 | 0.07 |
| Current smoker (%) | 10.2 | 6.9 | 0.02 |
| CVD history (%) | 6.7 | 6.9 | 0.91 |
| Body mass index (kg/m2) | 27.8 ± 5.3 | 27.5 ± 4.8 | 0.21 |
| Fasting glucose (mM) | 8.1 ± 2.5 | 8.0 ± 2.6 | 0.53 |
| HbA1c (%) | 7.75 ± 1.34 | 7.75 ± 1.29 | 0.96 |
| Heart rate (bpm) | 70 ± 11 | 71 ± 11 | 0.39 |
| Blood pressure (mmHg)  Systolic pressure  Diastolic pressure  Mean Arterial Pressure | 138 ± 18  79 ± 9  99 ± 10 | 140 ± 18  79 ± 9  99 ± 11 | 0.11  0.74  0.26 |
| Lipids profile (mM)  HDL Cholesterol  LDL Cholesterol  Triacylglycerol (IQR) | 1.30 ± 0.37  2.76 ± 0.81  1.37 (1.02- 1.91) | 1.29 ±0.35  2.74 ± 0.82  1.40 (1.03- 1.93) | 0.51  0.61  0.38 |
| Baseline PWV (m/s)  Follow-up PWV (m/s)  Change in PWV (m/s) | 7.0 ± 1.7  8.5 ± 2.4  1.16 (0.56-2.21) | 8.4 ± 2.3  6.9 ± 1.7  -1.04 (-2.03- -0.47) | <0.001  <0.001  <0.001 |
| Baseline renal function  eGFR (ml/min/1.73m^2^)  ACR (µg/mg) IQR | 89 ± 24  20.0 (6.00-83.0) | 88 ± 24  23.0 (6.00- 85.0) | 0.19  0.46 |
| Medications usage (%)  Statin  Insulin  RAS blocker  Calcium channel blocker  Beta blocker  Diuretics | 80.6  26.1  55.4  19.9  14.0  11.6 | 81.3  27.1  63.1  19.9  14.5  14.0 | 0.75  0.66  0.003  0.99  0.76  0.18 |

* Student t test, Mann-Whitney U test or χ2 test where appropriate. PP, pulse pressure; ACR, urinary albumin-to-creatinine ratio; RAS, renin-angiotensin system
